# Investigating the Cognitive Style of Patients With Substance Use Disorder: A Cross-Sectional Study

**DOI:** 10.7759/cureus.51800

**Published:** 2024-01-07

**Authors:** Savy Chawla, Syed S Kazmi, Akanksha Singh, Garima Singh

**Affiliations:** 1 Clinical Psychology, Gurukul The School, Ghaziabad, IND; 2 Clinical Psychology, Amity University Uttar Pradesh Lucknow Campus, Lucknow, IND; 3 Forensic Psychology, Amity University Uttar Pradesh Lucknow Campus, Lucknow, IND; 4 Department of Psychiatry, Balrampur Hospital, Lucknow, IND

**Keywords:** stress, coping, substance use disorder, cognitive style, cognition

## Abstract

Background

The causal attributions we make to the events in our lives reflect our Cognitive Style. The use of substances can be precipitated by stressful life events, and substance use can be a result of maladaptive coping to alleviate negative effects in stressful situations. So, individuals with substance dependence may infer situations differently. The inferences made about the cause of these stressful events can give an understanding of their cognition and can further help in therapeutic interventions.

Purpose

The present study aims to assess the cognitive style of young patients with substance use disorder.

Methods

A cross-sectional research design was used and a total of 50 participants were chosen through purposive sampling from the in-patient departments of Psychiatric Hospitals and De-addiction centers. The Alcohol, Smoking, and Substance Involvement Screening Test (ASSIST) was used to assess the specific substances used by the patients and the Cognitive Style Questionnaire-Short form (CSQ-SF) was used to assess the negative cognitive style of the patients.

Results

Results revealed a more negative cognitive style among young patients with Dual Substance Use than patients with Multiple Substance Use, indicating that patients with Substance Use Disorder tend to attribute stressful events to causes like *internal* (because of self), *global* (applicable to all domains of life) and *stable *(consistent), as well as the negative consequences (leading to other bad things) and self-worth implications (something wrong in self).

## Introduction

Cognitive Style is a person’s habitual, prevalent, or preferred way of thinking [1]. It is an individual’s approach applied while undergoing any cognitive task. It could be a stable indicator of the way of perceiving, interpreting information, and responding to the environment [2]. Cognitive styles are assumed to influence people's values, attitudes and social interactions and are considered a personality feature that represents both nature and nurture effects. Cognitive styles are the "lenses" through which individuals usually process information and interpret their reality and they may determine the cognitive vulnerability or cognitive risk of an individual to develop psychopathology over time [3]. Cognitive styles can prospectively predict the inferences one makes about the stressful events in one's life [4]. As compared to low-risk individuals, high-risk individuals are likely to draw negative inferences about the causes, consequences and self-worth implications of an event [5].

The consequences of these stressful life events may initiate or maintain Substance Use Disorder because the use of substances is frequently used as a coping method, despite their negative impact on one's ability to fulfill one's roles and responsibilities [6]. Substance use refers to the pattern of harmful or hazardous use of any psychoactive substance or drug, including alcohol and illicit drugs [7]. The use of psychoactive substances involves acute intoxication, harmful use, dependence, withdrawal state, psychotic condition, and amnesic syndrome as given by International Classification of Diseases-10 [8]. A study by Arora et al. (2016), revealed that 91% of the medical students engaged in substance abuse, were found to be aware of the ill effects of substance use and the most common reason of substance use was found to be psychological stress and occasional celebrations [3].

Continuous encounter with stressful events makes an individual look at things from a colored lens and give rise to negative attributions to the occurrences of events, thus making an individual stuck in the loop of disturbed cognitions. Emotional and cognitive styles were found to be the predictors of stress responses over time. Reduced use of cognitive reappraisal combined with stress, predicted an increase in alcohol consumption [9]. Attributional style differs significantly among drug-de-addicted relapsers and non-relapsers, wherein relapsers scored higher on the internality domain than non-relapsers [10].

Negative cognitive style is one such cognitive attribute, that reflects the way of thinking about stressful events that increases an individual’s risk for mental disorders like depression, after a stressful event [11]. A case-control study by Shakeri et al. (2021) [12], on patients with opioid use disorder, indicated a higher prevalence of cognitive avoidance as a coping mechanism than in the control group and Burnett et al. (2014) [5] indicated that males having external attributional styles engage in higher amounts of alcohol and drug use. People who draw negative conclusions from stressful events may attribute these events as stable and global, they see things as having widespread negative consequences and feel inadequate and hopeless about themselves, so consequently are more susceptible to depression [13].

Negative cognitive style was associated with greater depressive symptoms in undergraduate students [14]. This increased susceptibility to depression, in turn, makes the individual self-medicate and hide the depressed feeling by engaging in substance abuse, as it is the most prevalent form of maladaptive coping. Many individuals use substances to deal with their negative affective states due to the severity of their mental health problems. This suggests that most people with substance use disorder are vulnerable to developing other psychiatric disorders or vice versa. Prior literature has indicated profoundly that alcohol and tobacco use among adolescents indicated that substance use is associated with more physical and psychological symptoms, worse relationships with teachers and peers, less family support, and lower future expectations [6]. In a similar realm, a study conducted by Bravo et al. (2020) pressed on the relationship between negative effects and alcohol and marijuana use outcomes among dual users [4]. It was reflected that stress was indirectly related to alcohol and marijuana use, and depressive and anxiety symptoms were indirectly related to alcohol use only. All three negative affect symptoms were also indirectly related to negative consequences.

Therefore, substance use is considered a problem of altered cognition, not only from a neurological but also from a psychological perspective [15]. It has been indicated that patterns of alcohol use have a significant relationship with impulsivity and locus of control [16]. Relapses were more common in patients with a high external locus of control and impulsivity. To bridge the gap existent in the literature pertinent to cognitive style, the current study was undertaken. The present study aims to assess the cognitive style of patients with substance use disorder.

## Materials and methods

Objectives

To identify specific substances used by the patients with substance use disorder and to assess the cognitive style of the patients with dual substance use and multiple substance use.

Methodology

The study used the cross-sectional study design. The study population comprised individuals diagnosed with mental and behavioral disorders due to the use of psychoactive substances (according to the International Classification of Diseases (ICD)-10). Using the purposive sampling method 50 patients were selected from in-patient departments of psychiatric hospitals and de-addiction centers in Lucknow, Uttar Pradesh. Consenting and cooperative patients in the age range 18-45 years with minimum educational qualification till 8th standard who have been diagnosed with mental and behavioral disorders due to the use of psychoactive substances (according to ICD-10) by the treating psychiatrist/clinical psychologist were included in the study. Whereas, non-cooperative patients with a history of psychotic episodes or any other psychiatric or medical comorbidities were excluded. Patients in an intoxicated state or undergoing a withdrawal period were also excluded.

Data Collection 

After obtaining ethical clearance from the Departmental Research and Ethics Committee of the Department of Clinical Psychology, Amity University Lucknow, data collection was started. The semi-structured socio-demographic and clinical data sheet was designed by the researcher, particularly for the current research. The data sheet included information about sociodemographic and clinical details such as age, gender, place of residence, education, occupation, marital status, annual income, type of substances used, duration of substance, reason to start using substance, and presence of any medical or psychiatric illnesses. In order to identify the specific substances used by the patients, the Alcohol, Smoking, and Substance Involvement Screening Test (ASSIST) by the World Health Organization, 2010 was administered [17]. It is an eight-item measure of lifetime and current (past three months) use of 10 substances namely tobacco products, alcohol, cannabis, cocaine, amphetamine-type stimulants (ATS), sedatives, and sleeping pills (benzodiazepines), hallucinogens, inhalants, opioids, and other drugs. The ASSIST assigns a risk score to each substance. Each substance’s score falls into one of the three risk categories: low, moderate, and high, determining an appropriate intervention for that degree of use, i.e., no intervention, brief intervention, and intensive treatment respectively. It is culture-neutral, and the internal consistency reliability of the test is 0.70 to 0.95.

To assess the cognitive style of patients, the Cognitive Style Questionnaire-Short Form (CSQ-SF) by Meins et al. (2012) was used [18]. It consists of eight hypothetical scenarios in academics, achievement, employment, and interpersonal domains. These eight questions have nine different response items which are further scored on the five dimensions of cognitive style, i.e., Internality sub-scale, Globality sub-scale, Stability subscale, Negative consequences sub-scale, and Self- Worth Implications sub-scale. The participants rate the extent to which the cause of the events was internal (caused by something about self or something else), stable (the reason causing the same event to happen in the future), global (causing problems in other parts of life) as well as the consequences of the event (leading to other bad things in life due to the event) and self-worth implications (inferring to something wrong in self-due to the event). The scale has an internal reliability of 0.85 and a high face and construct validity.

*Statistical Analysis*

Data was collected from 50 male patients following the inclusion criteria using the semi-structured socio-demographic and clinical data sheet, ASSIST and CSQ-SF. The dataset collected through the questionnaires was analyzed for statistical outcomes using descriptive and inferential statistics including frequency, mean and t-test using Statistical Package for the Social Sciences (SPSS), version 20.0 (IBM Corp., Armonk, NY). The results of statistical analyses are elaborated further using adequate descriptions.

## Results

The results were evaluated using descriptive statistics (frequency distribution) and t-tests to know the significant difference in cognitive style of patients with substance use disorder. Table 1 displays the frequency and percentage of substances used and the type of user (dual substance or multiple substances) among patients with substance use disorder. The results showed that 50% (N=25) of the patients use a combination of two substances (dual substance users) and 50% (N=25) of the patients use a combination of more than two substances (multiple users).

**Table 1:** Description of clinical variables of patients with substance use disorder

Clinical variable		Frequency	Percentage
Substances used	Tobacco, Alcohol	25	50%
	Tobacco, Alcohol, Cannabis	10	20%
	Tobacco, Alcohol, Opioids	4	8%
	Tobacco, Cannabis, Opioids	3	6%
	Tobacco, Alcohol, Cannabis, Opioids	6	12%
	Tobacco, Alcohol, Cannabis, Inhalants	1	2%
	Tobacco, Alcohol, Cannabis, Inhalants, Opioids	1	2%
User	Dual	25	50%
	Multiple	25	50%

Table 2 depicts the scores of the cognitive style of the patients with substance use disorder. The mean and standard deviation of the total score on CSQ-SF is (194.40±21.95). The mean score on the Internality domain is (40.04±7.21); on the Globality domain is (39.62±4.99); on the Stability domain is (38.30±7.45); on the Negative Consequences domain is (22.30±5) and on Self-Worth Implications domain is (47.32±9.29).

**Table 2:** Cognitive style of patients with substance use disorder

	N	Minimum Score	Maximum Score	Mean	Standard Deviation
Cognitive Style	50	152	251	194.40	21.95
Internality	50	32	70	40.04	7.21
Globality	50	32	50	39.62	4.99
Stability	50	24	60	38.30	7.45
Negative Consequences	50	14	32	22.30	5.00
Self-Worth Implications	50	32	64	47.32	9.29

Table 3 shows the cognitive style of dual and multiple substance use. There was no significant difference found in the cognitive style of patients with dual and multiple substance use. No significant difference was found in the domains of cognitive style (internality, stability, globality, negative consequences, and self-worth implication) between dual and multiple substance users.

**Table 3:** Cognitive style of patients with dual substance use and multiple substance use

Variables	User	N	Mean	Standard Deviation	Standard Error Mean	t-value	p-value
Cognitive Style	Dual	25	194.64	20.82	4.16	0.07	0.93
	Multiple	25	194.16	23.45	4.69		
Internality	Dual	25	50.12	7.30	1.46	1.05	0.29
	Multiple	25	47.96	7.11	1.42		
Globality	Dual	25	39.96	5.35	1.07	0.47	0.63
	Multiple	25	39.28	4.68	0.93		
Stability	Dual	25	37.44	6.37	1.27	-0.81	0.42
	Multiple	25	39.16	8.43	1.68		
Negative consequences	Dual	25	21.64	4.77	.95	-0.93	0.35
	Multiple	25	22.96	5.23	1.04		
Self-worth implications	Dual	25	47.04	9.74	1.94	-0.21	0.83
	Multiple	25	47.60	9.02	1.80		

## Discussion

The present study aimed at exploring the cognitive style of the patient with substance use disorder. Substance use was assessed using the Alcohol, Smoking, and Substance Involvement Screening Test (ASSIST) and cognitive style was assessed using the Cognitive Style Questionnaire-Short Form (CSQ-SF). Further appropriate statistics were used for data analysis.

In the present study, the age of the patients was from 18 to 45 years with a mean age of 31.82 years. All the participants in the study were male. However, in the last decade, there has been a shift in observing substance use and abuse as an exclusive adult male phenomenon than in other populations [19].

With reference to the specific substance used by the patients, 50% of patients used alcohol and tobacco only and 50% of patients used multiple substances, i.e., three or more than three. It was found that 20% of patients used tobacco, alcohol, and cannabis, 8% of patients used tobacco, alcohol, and opioids, 6% of patients used tobacco, cannabis, and opioids, 12% of patients used tobacco, alcohol, cannabis opioids, 2% patients used tobacco, alcohol, cannabis, inhalants and remaining 2% patients used tobacco, alcohol, cannabis, inhalants, opioids. Use of more than one substance is on the rise and often underreported due to primary use of substance being attributed to the ‘high’ experience by the individual and to the withdrawal symptoms experienced on abstinence.

The present study reveals that half of the patients (50%) with substance use disorder had more negative cognitive style than others. Having a negative cognitive style makes an individual more cognitively vulnerable, thus leading to a high probability of developing depression-like features. In the domains of cognitive style, the majority of the patients (88%) scored more than average on internality, 48% of patients scored more than average on globality, 40% of patients scored more than average on stability, 46% patients scored more than average on negative consequences and on self-worth implications. This reveals that most of the patients tended to attribute the negative life events to more internal, global, and stable factors, which may be due to insufficiency in self and may lead to negative consequences in the future, affecting all areas of life. Liu et al. (2013) suggested in their study that negative cognitive styles may be crucial to be addressed in clinical settings, specifically in patients with a history of adverse childhood experiences, in order to reduce the incidence of negative life events, and hence the chance of depression recurrence [20]. The opposite effect was discovered in a study of explanatory style among community-dwelling older adults by Isaacowitz et al. (2003), wherein, adults with optimistic explanatory style (those who made external, temporary, and specific explanations for negative events) had most depressive symptoms at follow up [21].

However, the results show no significant difference in the cognitive style of the patients with dual substance use and multiple substance use. Therefore, the hypothesis that there will be a significant difference in the cognitive style of patients with dual substance use and multiple substance use, is not accepted. Patients with dual substance use were found to have a more negative cognitive style than those with multiple substance use but there was no significant difference in the cognitive style between the two groups. Supporting the finding, Debbie F (2007) found no considerable variation in the attributional styles between addicts and non-addicts [10]. Perez-Bouchard et al. (1993) revealed that variations in attributional style between children of substance abusers and children of nonsubstance abusers were predominantly due to the stability and globality components (i.e., stable and global attributions for negative occurrences) [22]. Hence, individuals having a pessimistic attributional style were more likely to relapse after a period of abstinence.

This study is a male-only study with a small sample size. The self-report data might be responsible for reporting bias, hence, the findings may not be generalized adding to the limitations of this study.

## Conclusions

The present study aimed at exploring the cognitive style of patients with substance use disorder. The findings of the study indicate that most of the patients with substance use disorder using tobacco, alcohol, cannabis, and inhalants have a negative cognitive style, whereas patients with dual substance use have a more negative cognitive style than patients with multiple substance use, i.e., they tend to attribute the life events in a more negative way. Assigning cause to a behavior or an event helps people understand themselves and learn to avoid engaging in more negative behaviors. Therefore, understanding the cognitive style of the patients can be helpful in therapeutic intervention.
